# Genome Editing and Its Applications in Model Organisms

**DOI:** 10.1016/j.gpb.2015.12.001

**Published:** 2016-01-04

**Authors:** Dongyuan Ma, Feng Liu

**Affiliations:** State Key Laboratory of Membrane Biology, Institute of Zoology, Chinese Academy of Sciences, Beijing 100101, China

**Keywords:** Genome editing, CRISPR/Cas, Zebrafish, Disease model, Gene therapy

## Abstract

Technological advances are important for innovative biological research. Development of molecular tools for DNA manipulation, such as zinc finger nucleases (ZFNs), transcription activator-like effector nucleases (TALENs), and the clustered regularly-interspaced short palindromic repeat **(CRISPR)/CRISPR**-associated **(Cas)**, has revolutionized **genome editing**. These approaches can be used to develop potential therapeutic strategies to effectively treat heritable diseases. In the last few years, substantial progress has been made in **CRISPR/Cas** technology, including technical improvements and wide application in many model systems. This review describes recent advancements in **genome editing** with a particular focus on **CRISPR/Cas**, covering the underlying principles, technological optimization, and its application in **zebrafish** and other model organisms, **disease modeling**, and **gene therapy** used for personalized medicine.

## Introduction

Since the discovery of the DNA double helix in 1953, many basic biological concepts pertaining to the genome, such as gene transcription and translation, genetic code and epigenetic modification, have been established by developing multiple experimental techniques. These include enzymes for *in vitro* DNA manipulations (such as polymerases, restriction endonucleases, and DNA ligases), recombinant DNA technology, *in vitro* DNA synthesis, site-specific mutagenesis, and whole-genome sequencing. Nonetheless, site-specific modification within genomes has remained a major challenge.

Genome editing, namely, refers to editing the nucleotides of the genome with engineered nucleases in cultured cells or living organisms. In the past decade, several types of engineered nucleases have been developed, including zinc finger nucleases (ZFNs), transcription activator-like effector nucleases (TALENs), and the recent clustered regularly-interspaced short palindromic repeat (CRISPR) systems. These nucleases, in particular CRISPR systems, immensely facilitate the wide application of genome editing in various biological research fields. More importantly, genome editing holds great promise in potential clinical applications such as gene therapy. In this review, we will briefly describe the features and development of these three editing methods and then mainly focus on the latest CRISPR technology, *Science*’s 2015 Breakthrough of the Year [1], and its application.

## ZFNs

ZFNs were discovered in 1996 and subsequently employed in genetic engineering experiments with *Drosophila* and mammalian cells in 2002 [2], [3], [4]. Unlike the previously reported approaches relying on DNA base-pair recognition, such as oligonucleotides, reverse splicing, or small molecules, the site-directed ZFNs act through DNA/protein recognition [2], [4], [5], [6], [7]. ZFNs are composed of a zinc finger-mediated DNA binding domain for DNA recognition and a nuclease activity domain of FokI for DNA cleavage [2]. ZFNs can cause double-strand DNA breaks (DSBs). Subsequently, insertion or deletion at the site of the genomic DSB can be induced by imprecise non-homologous end joining (NHEJ)-mediated repair, whereas point mutations or insertions from oligonucleotide or plasmid donor templates can be introduced by precise homology-directed repair (HDR)-mediated repair [8]. Over the past decade, ZFNs were optimized and widely used in, for example, targeted gene knockout in the genomes of mammalian cells to generate genetically distinct DHFR^−/−^ cell lines, establishing OCT4-eGFP human embryonic stem cells (hESCs) or targeting *PITX3* in induced pluripotent stem (iPS) cells, and heritable gene disruption in mouse and zebrafish [9], [10], [11], [12].

## TALENs

Plant pathogen *Xanthomonas* can secret TALEs upon infection of various host species, which facilitate bacterial infection or trigger defense by binding to promoter regions to activate effector-specific genes or R genes of the host plants [13], [14]. TALEs recognize specific DNA sequences via DNA-binding domains composed of nearly identical 34-amino acid repeated units. Two hypervariable amino acid residues at positions 12 and 13, named repeat-variable diresidues (RVDs), are required for target site specificity [15], [16]. Therefore, RVDs have been manipulated to generate the programmable DNA-binding proteins and used for site-directed genome editing [15], [16], [17], [18], [19]. Similar to ZFNs, the sequence-independent FokI nuclease, found in *Flavobacterium okeanokoites*, functions as the site-specific nuclease for TALEN assays when the target sites are recognized by different TALEs.

Due to the high similarity of TALE recognition sequences, a complicated procedure is required to generate programmable proteins that target specific sites on the genomic DNA, which limits the wide use of TALENs in genome engineering. The presence of extensive identical repeat sequences confers a huge technical challenge to clone repeated TALE arrays for different DNA target sites. To this end, several modified methods have been developed to enable rapid TALE assembly, including the ‘Golden Gate’ platform [20], [21], high-throughput solid-phase based sequential ligation systems [22], [23], and ligation-independent cloning techniques [24]. For target site recognition, it’s extremely important that the sequence of the TALE binding sites should start with a thymine (T). Moreover, the length of the target site and the spacer between the two TALEN arms are also very important for the formation of the FokI dimer and editing efficiency.

## CRISPR/Cas

CRISPR/CRISPR-associated (Cas) systems exist in prokaryotes to mediate bacterial adaptive immune defense against viruses or invading nucleic acids, as the first infection experiments showed that CRISPR/Cas confer resistance against lytic phages of *Streptococcus thermophilus* [25]. Brouns et al. found that mature CRISPR RNAs (crRNAs) work with Cas proteins to provide prokaryotes with antiviral defense by interfering with virus proliferation [26]. In 2012, Jinek et al. showed that the dual-RNA structure formed by crRNA and trans-activating crRNA (tracrRNA) is sufficient to direct *Streptococcus pyogenes* type II Cas9 protein (spCas9) to cleave specific target DNA sequences *in vitro* [27]. *In vitro* DNA cleavage by spCas9 and dual-RNAs reveals the potential of this system for genome editing. Subsequently, the RNA-guided editing tool for mammalian genomes was established using an engineered type II bacterial CRISPR system in 2013 [28], [29].

In the CRISPR/Cas system, crRNA–tracrRNA, also referred as the guide RNA (gRNA), recognizes the target sites on the genome, and then recruits Cas9 protein for precise cleavage at specific endogenous genomic loci [28], [29]. During this process, synthesis of the gRNA, composed of a specific 20-bp crRNA and the universal tracrRNA, can be driven by a U6 polymerase III promoter *in vivo* or by a phage RNA polymerase, such as T7 RNA polymerase, *in vitro* [29], [30], [31]. The first nucleotide of the gRNA target site should be a guanine (G) for U6-directed transcription and two guanines (GG) for T7-directed transcription [28], [29], [30], [31]. The most important region for target site selection by the CRISPR/Cas system is the protospacer adjacent motif (PAM) sequence, NGG, which mediates stimulation of the Cas9 nuclease activity [29]. Thus, compared to ZFNs and TALENs, the easy programmability of gRNAs is the most advantageous feature of CRISPR/Cas system (Table 1). Therefore, CRISPR/Cas has been quickly applied to generate mutations in different organisms, to establish various disease models, and for the use in gene correction and therapy [32].

## CRISPR/Cas in model organisms

The modified type II CRISPR/Cas, including the human codon–optimized versions of Cas9 and the specific gRNA, was first shown to work efficiently in HEK 293T cells, human leukemia K562 cell line, murine cell lines, and PGP1 iPS cells, using the *adeno-associated virus integration site 1* (*AAVS1*) or *empty spiracles homeobox 1* (*EMX1*) loci as target genes in February 2013 [28], [29]. Soon after, in March 2013, the synthesized Cas9 mRNA and gRNA targeting *fumarate hydratase* (*fh*) were shown to work *in vivo* to induce targeted genetic modifications in zebrafish as efficiently as ZFN and TALENs [33]. A month later, it was reported that Cas9/gRNA efficiently induced biallelic conversion of *etsrp* and *gata5* in zebrafish somatic cells and resulted in the abnormal intersegment vessels and cardia bifida, respectively, recapitulating the phenotype of *etsrp^y11^* and *fau^tm236a^* mutants described previously [34]. Later on, CRISPR/Cas-mediated gene editing was used to efficiently disrupt five genes simultaneously in mouse ESCs. Meanwhile, mice with biallelic mutations in *Tet1* and *Tet2* were generated by co-injecting Cas9 mRNA and gRNA targeting *Tet1* and *Tet2* into mouse zygotes in May 2013 [30].

**Figure 1:**
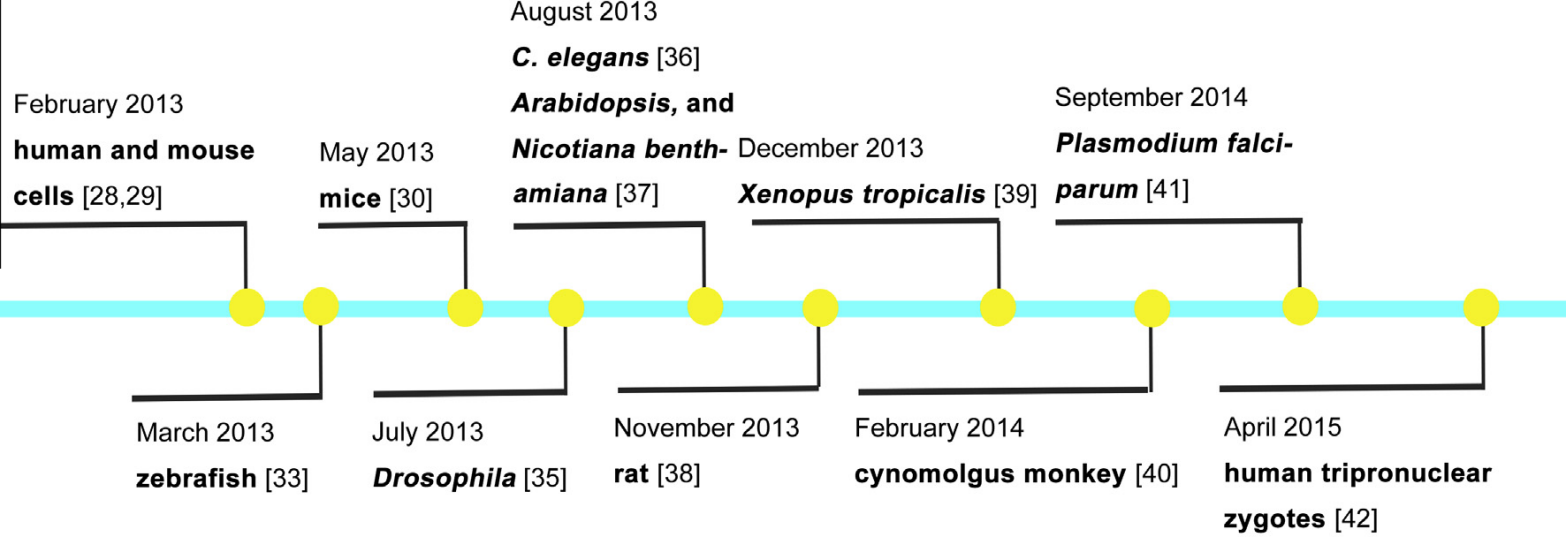
**The timeline for applications of CRISPR/Cas technology in model organisms**

In *Drosophila*, efficient mutagenesis of the *yellow* gene was induced by injecting *Drosophila* embryos with a single guide RNA (sgRNA) targeting the second exon of the gene, and animals carrying stable germline mutations were obtained [35]. CRISPR/Cas9 mediated heritable genome editing in *Caenorhabditis elegans* was established by Calarco’s group in August 2013. Expression of Cas9 protein, together with specific sgRNAs targeting the coding sequences of the *unc-119* and *dpy-13* genes, caused insertion or deletion (indels) in these two genes, and the resulting animals exhibited previously identified phenotypes, such as uncoordinated (Unc) and dumpy (Dpy) [36]. As shown in Figure 1, the CRISPR/Cas system has been rapidly utilized in an increasing number of model organisms, including *Arabidopsis* [37], *Nicotiana benthamiana* [37], rat [38], *Xenopus tropicalis* [39], cynomolgus monkey [40], *Plasmodium falciparum* [41], and even in human tripronuclear zygotes [42].

## CRISPR/Cas in zebrafish

Zebrafish (*Danio rerio*) serves as a classic model organism owing to its unique features, including external fertilization, transparent embryos, high fecundity, and rapid growth. Technologies for genome editing, such as ZFNs, TALENs and CRISPR/Cas, have been applied in zebrafish soon after they were initially reported (Table 2). *fh* was the first gene to be efficiently engineered in zebrafish using CRISPR/Cas [33], and then zebrafish-codon-optimized Cas9 was generated to improve the genome editing efficiency [31], [43]. Knock-in of DNA cassettes into the zebrafish genome using CRISPR/Cas9 was carried out by co-injecting a donor plasmid, together with the gRNA and a capped Cas9 mRNA, into one-cell stage embryos. With this convenient approach, specific zebrafish eGFP lines, including Tg(*neurod*:eGFP), Tg(*vsx2*:eGFP), and Tg(*pou4f3*:mGFP), were converted into Gal4 transgenic lines, which facilitated the creation of reporter or loss-of-function alleles in zebrafish [44].

**Figure 2:**
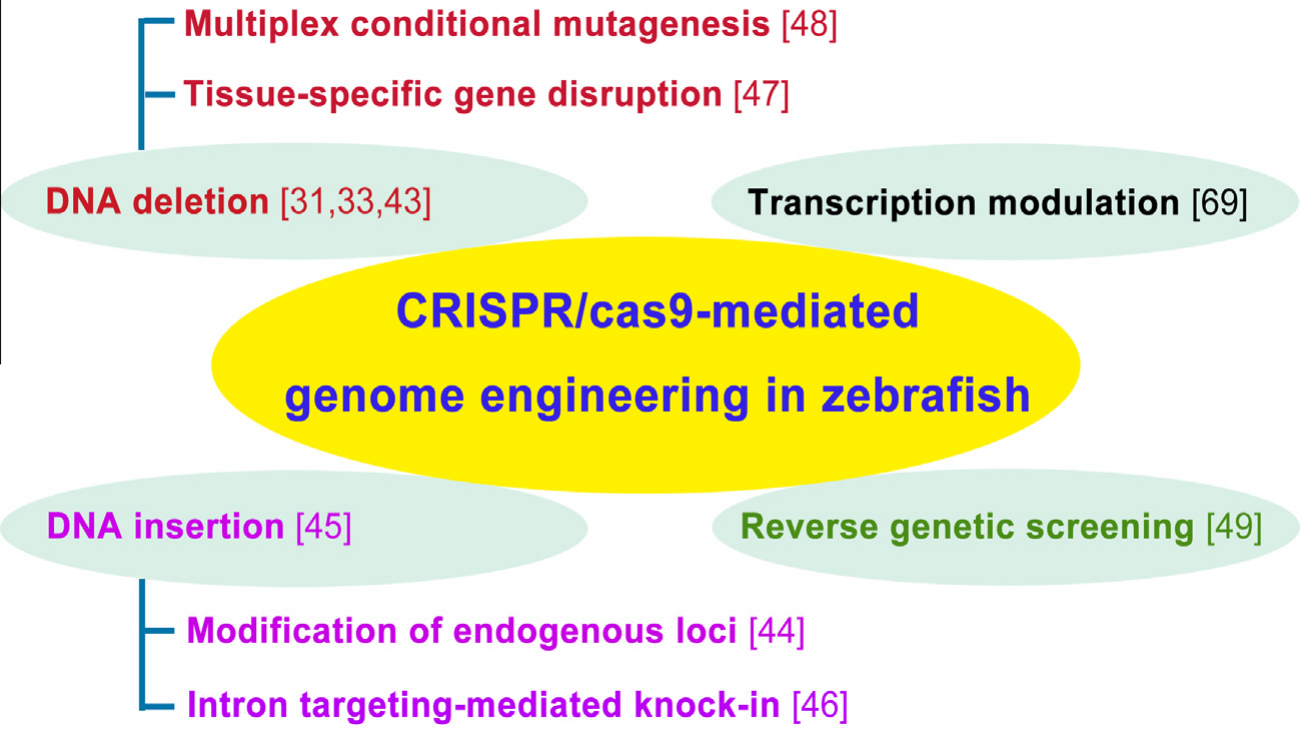
**CRISPR/Cas in zebrafish**

CRISPR/Cas9 was also used to facilitate an HA tag knock-in at the *C13H9orf72* (C9t3) locus using a donor oligonucleotide [45]. It was shown that P2A-EGFP was knocked-in to the endogenous zebrafish *tyrosine hydroxylase* (*th*) to trace *th* positive cells *in vivo* using an intron targeting-mediated and an endogenous gene integrity-maintaining strategy with the CRISPR/Cas system [46]. Zon’s group generated a CRISPR/Cas9 vector system for tissue-specific gene disruption and the *urod* gene was disrupted under the control of different tissue-specific promoters, mimicking human hepatic cutaneous porphyria in zebrafish [47]. Interestingly, multiplex-conditional CRISPR/Cas9-based mutagenesis in zebrafish can be achieved with Cas9 driven by the heat-shock-inducible or tissue-specific promoters and the Golden Gate assembly of sgRNA-expressing cassettes to allow temporally or spatially restricted gene inactivation [48]. In addition, CRISPR/Cas was used *in vivo* for rapid, reverse genetic screening of 48 loci in zebrafish. As a result, two new genes were demonstrated to be involved in electrical synapse formation [49]. Taken together, the application of CRISPR/Cas in zebrafish is becoming more popular, along with the modifications to this technology (Figure 2). In the meanwhile, zebrafish has become a useful model for technical improvement of CRISPR/Cas system.

## Technological improvement of CRISPR/Cas

So far, CRISPR/Cas has been used in most of the well-established model organisms. To improve the efficiency and specificity of the CRISPR/Cas system, different codon-optimized versions of Cas9 protein have been generated for different organisms, including human [29], mouse [30], [50], and zebrafish [31]. As *in vivo* genome editing in adult organisms is limited by the cargo size of the adeno-associated virus (AAV) vector, Zhang’s group identified shorter Cas9 from *S. thermophilus* LMD-9 (St1Cas9) [28] and *Staphylococcus aureus* (SaCas9). They went further to engineer these Cas9, together with gene specific gRNA, into a single AAV vector to target the cholesterol regulatory gene *Pcsk9* in the mouse liver [51]. They also characterized a new RNA-guided endonuclease named Cas protein 1 of PreFran subtype (Cpf1). Cpf1 utilizes a T-rich PAM and exhibits efficient genome-editing activity in human cells [52]. The studies from Zhang’s lab not only showed direct evidence for genome editing in mammalian cells, but also improved the efficiency, utility, and potential applications of the CRISPR/Cas system in broad research fields.

### Efficiency improvement via gDNA modification

CRISPR/Cas system was also optimized through gRNA modification. Chen’s group developed an optimized CRISPR/Cas system to achieve high rates of biallelic gene disruption in zebrafish F0 populations. Besides the zebrafish codon-optimized Cas9 protein, the sequence of the 3′-end of the crRNA::tracrRNA chimera was modified to GGAUC instead of a string of U residues normally found at the end of gRNA [31]. In following studies, it was found that the use of fewer than 20 nucleotides for gRNA complementarity could minimize off-target effects without sacrificing on-target genome editing efficiencies [53]. Recently, CRISPR subtype Ypest protein 4 (Csy4), an endoribonuclease from the bacterium *Pseudomonas aeruginosa*, was used to expand genome targeting sites in human cells and zebrafish [54], [55]. In addition, chemically-modified gRNAs were shown to increase the frequency of gene disruption in human primary T cells and CD34^+^ hematopoietic stem and progenitor cells (HSPCs) without evident toxicity [56]. With the codon-optimized Cas9, the shorter endonucleases, and the modified gRNA and expansion sites, CRISPR/Cas will become a common experimental technique for life science, just like gene cloning.

### Target specificity optimization

Point mutations in the two nuclease catalytic domains of Cas9, HNH and RuvC, can convert Cas9 into a DNA nickase, which is called Cas9 nickase [27], [28]. Moreover, using double Cas9 nickases in combination with the sgRNAs targeting opposite DNA strands of the target sites can cause DSBs with low off-target activity [57]. Quantitative analysis showed that Cas9 nickase could reduce off-target activity by 50–1500 folds in various cell lines [57]. The high target specificity of Cas9 nickase was further verified by Skarnes’ group. They found no detectable NHEJ-induced damage at the reported off-target sites recognized by wild-type Cas9 endonuclease both in mouse embryos and cultured cells [58]. Another successful example for SpCas9 mutant is the recently-reported “enhanced specificity” SpCas9 (eSpCas9) variants generated based on structure-guided protein engineering. eSpCas9 can maintain on-target efficiency and exhibit improved specificity [59].

Similar to ZFNs and TALENs, the smart combination of the inactive Cas9 protein (dCas9) and FokI nucleases was developed by two groups at the same time [55], [60]. In the FokI-based CRISPR/Cas system, the FokI nuclease domain is fused to a catalytically-inactive Cas9 protein. After being recruited by two gRNAs, the dimers of the FokI fusion protein mediate sequence-specific DNA cleavage, with a defined spacing and orientation [55], [60]. Quantitatively, the specificity of the FokI-based CRISPR/Cas was at least 140 fold higher than that of the wild type Cas9, and even fourfold higher than that of Cas9 nickase at similar endogenous off-target loci [60]. Recently, it was shown that SpCas9 can be modified with altered PAM specificity in zebrafish embryos and human cells. The specificity of a SpCas9 variant containing non-canonical NAG and NGA was increased in human cells [61]. In summary, both Cas9 nickase and FokI-based CRISPR/Cas can improve DNA cleavage specificity with lower off-target activity, which makes highly specific genome-wide editing much easier.

### Inducible CRISPR system

Conditional mutagenesis is often necessary to uncover the mechanism of gene function. Efforts to modify the efficiency and specificity of the CRISPR/Cas system also involve control of Cas9 nuclease activity in a spatial and temporal manner. Firstly, the inducible CRISPR (iCRISPR) system, composed of doxycycline-regulated Cas9 and a specific gRNA, was developed for genome editing in human pluripotent stem cells (hPSCs) and adult mice, and for generation of stage-specific inducible gene knockouts [62], [63], [64]. In addition, low copy expression of the rapamycin-inducible split-Cas9, composed of Cas9(N)-FK506 binding protein 12 (FKBP) rapamycin binding (FRB) and Cas9(C)-FKBP, can induce mutations at *EMX1* loci, while split dCas9-VP64 can mediate inducible transcription activation in HEK293FT cells [65]. Furthermore, photoactivatable Cas9 (paCas9) that responds to blue light irradiation was generated on the basis of split-Cas9 and photo-inducible dimerization domains named Magnets. paCas9 has been exploited and validated for efficient genome editing in human cells [66]. The greatest advantage of this system is its spatiotemporal and reversible feature, making it a potential alternate to the Cre-loxP system in generating conditional knockouts *in vivo*. Moreover, paCas9 and specific gRNAs targeting different endogenous genes may facilitate multiple gene knock-outs *in vivo* in a spatiotemporal manner, which would be much easier than other systems.

### Gene regulation by dCas9 fused with effector domains

In addition to genome editing, a modified form of Cas9 that lacks the endonuclease activity, dead Cas9 (dCas9), was first shown to regulate endogenous gene expression in *Escherichia coli* and mammalian cells [67]. This system, called CRISPR interference (CRISPRi), can repress the expression of targeted genes. Soon after, dCas9 was fused to effector domains with distinct regulatory functions for stable and efficient repression or activation at the transcriptional level in human and yeast cells [68]. The specificity of CRISPRi-mediated transcriptional regulation was determined solely by the co-expressed short gRNA that can recognize the endogenous target gene. dCas9-KRAB or dCas9-VP160 was used to alter expression of the endogenous *dpy-5* and *dbl-1* in *C. elegans* or *fgf8a* and *foxi1* in zebrafish embryos, respectively [69]. More importantly, the modified photoactivatable dCas9 (padCas9) system can reversibly control spatiotemporal expression of endogenous genes [66].

Cas9 is an RNA-guided DNA endonuclease, which requires a short DNA sequence named PAM for binding and catalysis. It was found, however, that Cas9 can also bind to single-stranded RNA (ssRNA) in the presence of specially-designed PAM-presenting oligonucleotides (PAMmers) and a matching gRNA, leading to site-specific cleavage of ssRNA targets while ignoring the corresponding DNA sequences [70]. In addition, dCas9 with specifically designed 5′-extended PAMmers and gRNA can target non-PAM sites on *GADPH* mRNA, allowing this mRNA to be purified from HeLa cells in a tagless manner [70]. Therefore, programmable RNA recognition by CRISPR/Cas can be used as a candidate approach for specific endogenous mRNA isolation, analysis, and manipulation in the absence of affinity tags [70]. The dCas9 not only expands the application of CRISPR/Cas to areas other than genome editing, but also provides a candidate method for spatiotemporal and reversible gene regulation *in vivo*, with the help of padCas9 controlled by blue light irradiation.

## Generation of disease models using CRISPR/Cas

In addition to site-specific modifications in the genomes, TALENs and CRISPR/Cas also have potential applications in disease models and gene correction. First, human stem cell-based disease models were generated using TALENs, and different disease-related genes were analyzed, including *APOB* for human hepatitis C virus (HCV) replication, *SORT1* (encoding sortilin) for ApoB secretion in hepatocytes, insulin resistance in adipocytes and motor neuron death, and *PLIN1* for lipolysis in adipocytes [71]. More importantly, heritable disease models have been easily generated. For instance, lentivirus-delivered sgRNA:Cas9 genome editing was used to generate mouse models of myeloid malignancy by modifying five genes in mouse hematopoietic stem cell (HSC) [72]. And a cancer model was also generated using the CRISPR/Cas system by targeting tumor suppressor genes *pten* and *p53* in liver [73]. As for zebrafish disease models, a *rps19* null mutant was generated using TALENs to reproduce the erythroid defects of Diamond–Blackfan anemia (DBA) [74]. Recently, human hepatic cutaneous porphyria was mimicked by a tissue-specific gene inactivation system resulting from disruption of the *urod* gene using a different tissue-specific promoter [47].

Using CRISPR/Cas system, Liu’s group generated a series of mutations in the blood development related genes of zebrafish, including *ncor2* [75], *runx1*, *runx3*, *rac2*, and *klf6a* (unpublished data), that could be used to model human hematological diseases, including bone marrow failure, anemia, and myelodysplasia syndrome (MDS). The Zebrafish All Genes KO Consortium for Chromosome 1 (ZAKOC) has been established with the efforts of nearly all the zebrafish labs in China since June 2013. This project would accumulate zebrafish mutant resources for basic sciences and also for disease modeling.

## Gene therapy on clinical mutations and disease models

Gene therapy, based on therapeutic delivery of nucleic acid polymers into patient, is an innovative method that has generated much controversy. Development of CRISPR/Cas and TALENs makes gene therapy more feasible and easier in the treatment of diseases. CRISPR/Cas was firstly used for efficient correction of disease-related genes in mouse and intestinal stem cells of a cystic fibrosis (CF) patient at the end of 2013 [76]. Clevers’s group cultured intestinal (LI) stem cells from CF patients homozygous for the most common cystic fibrosis transmembrane conductance regulator (CFTR) mutations and used CRISPR/Cas-mediated homologous recombination to correct the *CFTR* locus. Their data indicated that the corrected allele could be detected and was confirmed to be functional [77]. A single nucleotide deletion in exon 3 of the *Crygc* gene in mice leads to the generation of a stop codon at the 76^th^ amino acid residue. Mice with the resulting dominant gene mutation serve as a model of dominant cataract disorder [76], [77]. Li’s group showed that the mutation in *Crygc* can be corrected at the organismal level through HDR, by co-injecting Cas9 mRNA, together with a specific gRNA targeting the mutant allele with exogenously-supplied oligonucleotide, into zygotes [76]. Further analysis showed that the mouse carrying the corrected mutation was fertile and able to transmit the modified DNA sequence to its progeny [76]. To date, there are a number of disease models reported to be corrected successfully using CRISPR/Cas-mediated homologous recombination, such as the *Fah* mutation in mouse hepatocytes [78] and the *mdx* mutation in the mice model for Duchene muscular dystrophy (DMD) [79].

β-thalassemia, caused by mutations in the adult β-globin gene, is one of the most common genetic diseases worldwide [80]. Pan’s group efficiently generated integration-free β-thalassemia iPS cells from the cells of patients and corrected β-globin gene (*HBB*) mutations *in situ* using TALENs with a donor template harboring the entire wild type β-globin gene [80]. Further study showed that the gene-corrected β-thalassemia iPS cell lines from each patient had restored *HBB* gene function in β-thalassemia-iPSC-derived hematopoietic progenitor cells (HPCs) and erythroblasts [80]. Later on, the *HBB* mutations in patient-derived iPSCs was also efficiently corrected using CRISPR/Cas9 combined with the *piggyBac* transposon without leaving a residual footprint. The corrected iPS cells were differentiated into erythroblasts with restored expression of *HBB* [81]. Fanconi anemia is another blood disease in which gene correction was attempted. The FANCC c.456 + 4A > T mutation in patient-derived fibroblasts was corrected using CRISPR/Cas9 with a donor plasmid containing a floxed puromycin and FANCC cDNA flanked with arms that were homologous to the FANCC locus [82].

## Conclusion and perspective

New tools for DNA manipulation, including TALENs and CRISPR/Cas, not only make site-specific modifications in the genomes much easier, but also revolutionize the classical approaches for determining gene function due to their target site specificity, flexible design, and ease of operation. Traditional forward genetics approaches can generate mutants with the desired phenotypes; however, the subsequent gene mapping is very complicated, and it is not feasible to perform large-scale genetic screens in some model organisms. Reverse genetics by gene targeting usually employs ESCs, which are limited to a few model systems, and the process is time-consuming. The newly-emerging technologies for genome editing can, in principal, target any gene of interest; therefore, specific modifications or targeted gene knockouts can be easily obtained in model organisms. So far, CRISPR/Cas has been quickly optimized and applied to most of the current model systems, and even in the human tripronuclear zygotes (Figure 1).

The CRISPR/Cas system has been continuously improved from different angles, including efficiency, specificity, spatiotemporal control, and endonuclease inactivation (Table 3). Until now, the CRISPR/Cas system not only improves basic research but also has potential applications in gene therapy through gene repair, gene disruption, and programmable RNA targeting [32]. However, there are also some disadvantages and controversial issues, for example, the off-target effect. To solve this problem, the chemically-modified gRNAs, Cas9 nickase, FokI-based CRISPR/Cas, and eSpCas9 can be harnessed to reduce off-target and improve the specificity.

For the clinical application of CRISPR/Cas in future, many points should be considered. The first question is how to guarantee the efficiency. In spite of the improvements on Cas9 and the gRNAs, the low efficiency of HDR after Cas9-mediated DNA cutting is a challenge. Importantly, it has been shown that inhibiting NHEJ-mediated repair enhances HDR for the insertion of precise genetic modifications by the inhibitor, Scr7 targeted DNA ligase IV, or by key molecules involved in gene silencing [83], [84]. The next question is how to deliver Cas9 and gRNAs to cells, especially to adult tissues. Fortunately, the shorter form of Cas9 in the AAV vector, nucleofection, cell-penetrating peptides, and the inducible Cas9 have been developed, which will provide candidate approaches for gene therapy in adult [85], [86]. Finally, the most controversial question is whether CRISPR/Cas can be used in humans ethically and safely. As Jennifer Doudna, a pioneer of CRISPR/Cas technology, pointed out, that with the rapid development of this technology and its wide application, the philosophical and ethical ramifications for altering genomes, especially on the modification of human germ cells and embryos should be very cautious, and an international guideline on the proper use of genome editing should be abided by the entire field [87], [88]. Overall, this ethical issue needs to be addressed by scientists and society before CRISPR/Cas can be used in clinical gene therapy and that CRISPR/Cas system also needs to be optimized on various aspects to improve the efficiency and specificity.

## Competing interests

The authors have no conflicts of interest to declare.

## Figures and Tables

**Table 1:** Comparison of ZFN, TALEN, and CRISPR/Cas

**Technology**	**Working mechanism**	**Essential components**	**Efficiency**	**Criteria for target site**	**Refs.**
ZFN	DNA/protein recognition	ZFN with zinc finger domain and FokI endonuclease domain	Variable	Preferential binding sequence for zinc finger proteins or Cys_2_His_2_ fingers	[2], [3], [4]
TALEN	DNA/protein recognition	TALE and FokI fusion protein	High but variable	TALE binding sites should start with a T with the space between two TALEN arms better 15–21 bp	[15], [16], [17], [18], [19]
CRISPR/Cas	DNA/RNA recognition	Cas9 protein and gRNA	High but variable	gRNA target site should be 20 bp long starting with a G for U6-directed transcription and GG for T7-directed transcription; PAM sequence (NGG) is indispensable for Cas9 nuclease activity	[28], [29], [30], [31]

*Note:* ZFN, zinc finger nuclease; TALEN, transcription activator-like effector nuclease; CRISPR, clustered regularly-interspaced short palindromic repeat; Cas, CRISPR-associated; gRNA, guide RNA; PAM, protospacer adjacent motif.

**Table 2:** Genome editing firstly reported in various biological systems and zebrafish

**Technology**	**First report on genome editing**				**First report on zebrafish**		
**Gene/locus**	**Species/cell line**	**Time**	**Ref.**	**Gene**	**Time**	**Ref.**
ZFN	*Yellow (y)*	*Drosophila*	Jul, 2002	[3]	*gol*	Jun, 2008	[11]
TALENs	*NTF3* and *CCR5*	HEK 293, K562	Feb, 2011	[17]	*tnikb*	Aug, 2011	[19]
CRISPR/Cas	*AAVS1* locus	HEK 293, K562, and PGP1 iPS cells	Feb, 2013	[29]	*tia1l* and *gsk3b*	Mar, 2013	[33]
*EMX1*	HEK 293	Feb, 2013	[28]			
*Th* loci	N2A	Feb, 2013	[28]			

*Note:* ZFN, zinc finger nuclease; TALEN, transcription activator-like effector nuclease; CRISPR, clustered regularly-interspaced short palindromic repeat; Cas, CRISPR-associated; NTDF3, neurotrophin-3; CCR5, chemokine (C–C motif) receptor 5; AAVS1, adeno-associated virus integration site 1; EMX1, empty spiracles homeobox 1; Th, tyrosine hydroxylase; gol, golden; tnikb, TRAF2 and NCK-interacting protein kinase; tia1, T-cell-restricted intracellular antigen-1; gsk3b, glycogen synthase kinase 3 beta.

**Table 3:** Optimization of CRISPR/Cas9 system

	**Cas9**		**gRNA**	
**Improvement**	**Refs.**	**Improvement**	**Refs.**
Efficiency	Codon-optimized Cas9	[29], [31], [50]	Modification on 3′ end of crRNA:tracrRNA chimera	[31]
St1Cas9 and SaCas9	[28], [51]	Csy4-based gRNA cleavage	[54], [55]
Cpf1	[52]	Chemically-modified gRNAs	[56]

Specificity	Cas9 nickase	[57], [58]		
eSpCas9	[59]		
FokI-based CRISPR/Cas	[55], [60]		

Cas9 activity	dCas9-KRAB and dCas9-VP160	[69]		
Photoactivatable dCas9	[66]		

Spatiotemporal control	Specific promoter driven Cas9	[47]		
Doxycycline-regulated Cas9	[62], [63], [64]		
Rapamycin-inducible split-Cas9	[65]		
Photoactivatable Cas9	[66]		

*Note:* Cas9, CRISPR-associated protein 9; St1Cas9, *Streptococcus thermophilus* Cas9; SaCas9, *Staphylococcus aureus* Cas9; Cpf1, Cas protein 1 of PreFran subtype; eSpCas9, “enhanced specificity” *Streptococcus pyogenes* type II Cas9; dCas9, catalytically inactive Cas9; KRAB, Krüppel associated box; gRNA, guide RNA; crRNA, CRISPR RNA; tracrRNA, *trans*-activating crRNA; Csy4, CRISPR subtype Ypest protein 4.
